# What Do Polish Parents and Caregivers Think of Dietary Supplements for Children Aged 3–12?

**DOI:** 10.3390/nu12103076

**Published:** 2020-10-09

**Authors:** Agnieszka Piekara, Małgorzata Krzywonos, Monika Kaczmarczyk

**Affiliations:** Department of Bioprocess Engineering, Wroclaw University of Economics and Business, Room 301H, Komandorska 118/120, 53-345 Wrocław, Poland; malgorzata.krzywonos@ue.wroc.pl (M.K.); monika.kaczmarczyk@ue.wroc.pl (M.K.)

**Keywords:** dietary supplement, supplements intake, children diet, attitude

## Abstract

Background: The aim of this study was to specify the amounts and the types of dietary supplements (DS) taken by children and define the attitudes of caregivers towards DS and towards administering them to children aged 3–12. An analysis of the reasons for using DSs, and of expected benefits and awareness of health risks associated with administering DSs, was conducted. Methods: The online questionnaire collected information on sociodemographic characteristics and use of dietary supplements. Multiple analyses were used to describe the relationship between demographic factors and dietary supplement intake. In particular cases, strength and correlation were also calculated. Results: In total, 54.89% of participants were administering dietary supplements to children at the time of completing the questionnaire—a weak linear relationship (Cramer’s V = 0.21) between child’s age and the child taking dietary supplements. Respondents for whom DSs are equivalents of medicines tend not to see that dietary supplements may cause side-effects and interact. Conclusions: Parents who administer dietary supplements to children show a tendency to have more trust in this type of product than the people who do not do so. It was also confirmed that the people who take dietary supplements transfer their behavioral patterns by also administering them to their children.

## 1. Introduction

The quality of diet in childhood has an enormous influence on the condition of the human body in adulthood. Providing optimal quantities of macro- and micro-elements is possible when following the principles of good nutrition. However, one can observe the growing importance of dietary supplements in the day-to-day diet of children as well as adults [1,2,3]. FDA defines dietary supplements as products (other than tobacco) intended to supplement the diet which bear or contain one or more of the dietary ingredients [4]. The European Commission defines them as foodstuffs the purpose of which is to supplement the normal diet and which are concentrated sources of nutrients or other substances with a nutritional or physiological effect [4,5]. This relatively new to the market type of products is growing in importance. An enormous variety of dietary supplements results in a growing interest in analyzing their intake (reasons, knowledge, attitudes, and practices) [6,7,8,9]. About 4.4% or even more of the supplement market is aimed at children [10]. Until now, DS intake among children was not a subject of a lot of research [11,12,13,14,15] unlike pregnant women, sportsmen, dieticians, and pharmacists [9,16,17,18,19].

It is essential from the social standpoint to get to know the volume of dietary supplements consumption and the type of products that are chosen by children’s caregivers. It is important because supplements available on the market may be mislabeled, others adulterated and some may even lack the bioactive ingredients promised by the producer’s declaration [20,21]. There may also be deviations between particular product series [22]. In the course of the study conducted by the National Institute for Public Health and the Environment in the Netherlands, some products were identified as having higher or lower quantities of vitamin D in comparison to the information included on the packaging of the products and which were intended for adults as well as children [23]. A similar case was described by Arakai [24], who concluded that the actual quantity of vitamin D in the recommended dose was 1000 times higher than the information on the product label indicated. It creates a threat of exceeding the upper level intake (UL) of this ingredient, which in the case of children, may lead to hypercalcaemia, hypercalciuria and kidney problems [25,26]. Children are a special group of potential recipients of supplements due to the fact that:

- They do not make their own purchasing decisions;
- They do not make their own decisions concerning diet [27,28,29];
- They fall victim to unfair marketing practices [30,31];
- They have different metabolic processes, which are responsible for biotransformation of active substances (metabolism and elimination), or various ways of the organism reacting to the used preparation [32,33,34];
- They have very varied dietary requirements [35].

As mentioned previously, not much research has been conducted on the subject of children taking dietary supplements, as well as about the Polish population of children aged 3–12 in the context of the amounts and the types of dietary supplements taken by them. The research that has been conducted until now did not discuss the formulations of supplements taken by children, which is a crucial parameter vis-a-vis the variety of the supplement formulations available on the market (especially the formulations, which take the form of conventional sweets), preferences of children and their caregivers as well as adjusting the formulation to suit the consumption capabilities of the target groups. Additionally the paper aimed to define the attitudes of caregivers towards SDs and towards administering them to children aged 3–12. An analysis of the reasons for using DSs, expected benefits and awareness of health risks associated with administering DSs was conducted.

## 2. Materials and Methods

A questionnaire was created to test the knowledge, practice, and attitudes of parents with regard to dietary supplements and especially sweetening agents and labeling of these products. The sample of the study was limited to parents and caregivers of children from Wrocław (Poland) and its suburbs (population of 600,000). Between June and September of 2017, an online survey was advertised through a mailing list by school and preschool principals and forums for parents and caregivers of children. Information obtained from respondents was held in confidence. Hence, the survey did not ask for names or any other personal details. To guarantee questionnaire validity and reliability, a structured questionnaire was developed after careful review of the literature. Validation was conducted by pre-testing on a group of 10 parents and caregivers of children aged 3 to 12 (diverse in terms of age, education, wealth, and gender) to limit the risk related to the potential lack of understanding of questions by respondents and rejecting irrelevant statements.

The questionnaire intended for parents and caregivers of children was divided into 4 parts. The first part was devoted to questions concerning sociodemographic features related to age, gender, education, place of residence and material status of the respondents. The second part of the questionnaire was dedicated to questions that enabled us to characterize children. It asked questions related to their age, gender, health status, diet, allergies and food intolerances. The third part was devoted to the respondent’s knowledge of dietary supplements, their efficacy, possible threats; attitudes and behaviors. The last part of the questionnaire was intended only for the respondents who administer dietary supplements to their children and included questions referring to attitudes, and behaviors related to administering dietary supplements to children.

### 2.1. The Study Participants Group

The sample size was measured using the Raosoft online calculator [8]. According to the Statistical Office in Wrocław as of 31 of December 2016, the number of children aged 3–12, living in Wrocław and Wrocław County, amounted to 75,146. In order to carry out the research, it was essential for at least 383 respondents to take part (a significant segment of the children’s population lives in families that have two or more children). However, for the sake of the calculations, data from the Statistical Office were used. The study sample (*N* = 383) was calculated on the assumption that the confidence level: 95%, margin of error: 5%.

### 2.2. Statistical Calculations

The nonparametric chi-square test (χ^2^) was used to examine the statistical significance of differences between variables expressed on the nominal scale. The variables are presented on the nominal scale when they take value for which there is no order resulting from the nature of this particular phenomenon. The non-parametric Mann–Whitney U test was used to examine the statistical significance of the differences between dependent variables that were expressed on the ordinal scale. To examine the statistical significance of the differences between sociodemographic features, characterized by two or more variables, and the dependent variables expressed on the ordinal scale, the nonparametric version of the Kruskal–Wallis one-way analysis of variance was used. Besides the assessment of the relevance of the relationship, the strength and correlation were also calculated. The statistical tool used to determine the strength of the relationship between nominal variables was the Crammer’s V based on chi-squared characteristics. Analysis of variables expressed on the ordinal or interval scale (when the unit of measurement can be determined) was conducted using the Spearman’s rank correlation coefficient. The results of the survey were subjected to statistical analysis using PQStat (1.6.4 version) and Statistica (12 version) statistical software.

## 3. Results

While conducting the research, the questionnaire was completed by 540 people. Eight questionnaires were not taken into consideration because unreliable data were provided. The group consisted of 532 participants, 90.98% of whom were female, and 9.02% male. The majority of the respondents (68.23%) were in the 31–40 age range. Most respondents had higher education and declared themselves to have a good or very good material situation (Table 1).

**Figure 1:**
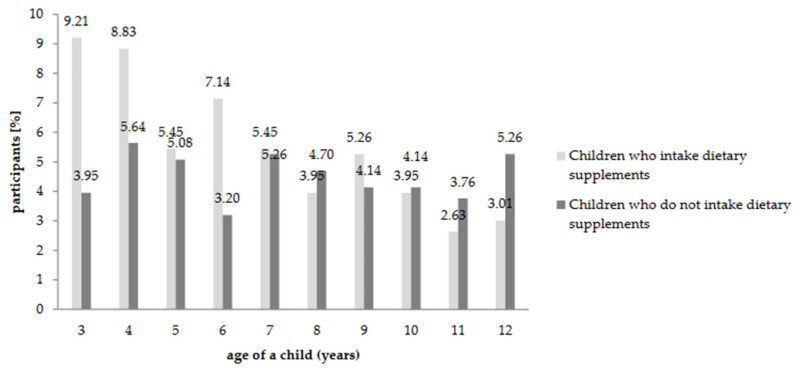
Relationship between child’s age and the child taking dietary supplements.

The sample group consisted of both users (40.04%) and non-users (59.96%) of dietary supplements. Moreover, out of the entire population: 54.89% were administering dietary supplements to children at the time of completing the questionnaire; 19.17% claimed that the child’s diet is balanced and there is no need for additional supplementation; 8.08% did not know whether the child’s diet is balanced, but believed that supplementation is not necessary; 9.40% did not trust dietary supplements; 6.39% indicated that children do not need such products; and for 2.07%, such products were too expensive. Based on the chi-square independence test (*p* ≤ 0.05) it was determined that there is a statistically significant relationship between respondents (parents and caregivers), who take dietary supplements and administered them to children. An important fact indicated by the respondents (*N* = 532) was that 94.55% of them had some experience in administering DSs to children. Important statistical differences were observed on the basis of the research (chi-square, *p* ≤ 0.05) along with a weak linear relationship (Cramer’s V = 0.21) between the child’s age and the child taking dietary supplements (Figure 1).

The majority of the respondents described their children’s health as very good (64.66%) or good (32.89%), and only 2.44% admitted that it is average. None of the respondents defined it as bad. The vast majority, that is, 87.03% of respondents, claimed that their children have a normal BMI (Body Mass Index), while 5.83% of children were described as overweight or obese, 6.02% underweight, and about 1% of respondents could not determine this parameter.

Almost 80% of children from the studied population ate meals in educational institutions. The menu was checked at least once a week by 45.11% of the respondents, and 25.75% of the respondents checked it very rarely or not at all. The individuals who administer dietary supplements to children tend to keep a closer eye on the meal plan (chi-square, *p* ≤ 0.05).

**Figure 2:**
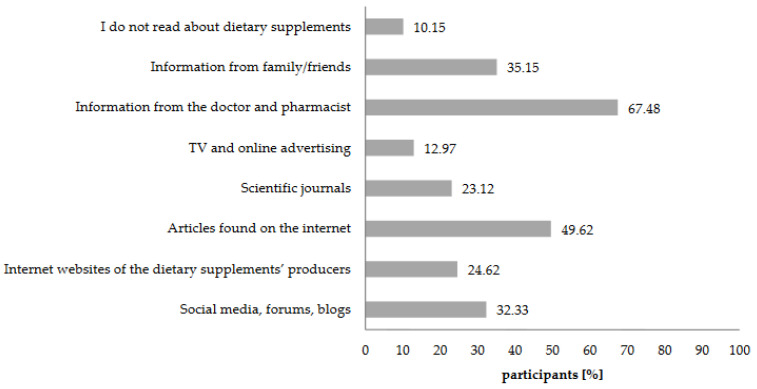
Sources of consumers’ knowledge on the subject of dietary supplements. Values do not add up to 100%, because more than one answer was allowed.

Doctors and pharmacists, as well as the materials originating from the online sources, were the primary sources of knowledge on the subject of dietary supplements among the respondents (Figure 2). Moreover, a significant number of respondents (81.51%, *n* = 292) sought the opinion of the specialist, i.e., doctor or pharmacist.

Respondents have also claimed that the decision to administer dietary supplements to children should only be consulted with a doctor (40.23%), or alternatively with a doctor or a pharmacist (40.79%), while 5.45% consulted their decisions with dieticians, and almost 6% consider that no consultations are necessary or they do not have an opinion (7.71%). The majority of the respondents (90.41%, *n* = 292) also informed a pediatrician about introducing supplementation into the child’s diet.

Respondents were asked about their understanding of the term “dietary supplement”. They had the freedom to define this term on their own. On the basis of the analysis of the responses, a division was made into three categories comparing dietary supplements to pharmaceuticals, food, and other neutral responses (Table 2). Among all of the responses, those that dominated (26.88%) equated dietary supplements with vitamins and/or minerals. The second largest group (25.94%) defined dietary supplements as preparations used to supplement the diet. Another noticeable group perceived supplements as drugs.

Evaluation of the knowledge and attitudes of the research participants was also conducted based on the answers they gave to 11 statements (Table 3). People who administered dietary supplements to children declared themselves to have a higher level of knowledge, whereby this group of respondents, as well as people not administering DSs, indicated that supplements are ‘probably’ not a type of food (median equals 2). On the basis of the accepted level of significance *p* ≤ 0.05 and the Mann–Whitney U test, it can be assumed that there are statistically significant differences between people administering and not administering DSs to children in the context of their attitude to the studied group of products. The first group shows a more positive attitude towards supplementation, also by declaring to have a higher level of general knowledge on the subject of dietary supplements, whereas the second group (not administering DSs to children) agrees to a much lesser degree with the statements indicating the possibility of supplementing the diet and therapeutic effects of DSs. This group also indicated more concerns in regards to the possible side effects and safety of such products (which was confirmed by lower numbers of answers to statements 4 and 5 in this group of respondents). The whole studied population indicated a high knowledge and awareness of possible DS–medication or DS–food interactions, and the dangers of adverse and toxic effects of DS after exceeding the recommended dose.

Among the answers, some referred to potential medicinal qualities of DSs (statement 9, 10, 11, Table 3) to get to know the attitudes (the perceptions) of consumers towards this group of products. It is worth mentioning that dietary supplements neither treat nor prevent diseases- they are foodstuff, i.e., food. However, the opinion of the sample group on this matter was different, 36.84% of the respondents (*N* = 532) believe that DSs can display medicinal properties. Almost 50% of the study participants group (as well as 62.33% of people administering dietary supplements to children *n* = 292) believed that they are successful in cold and flu prevention. The statement saying that administering dietary supplements to children during illness can shorten its duration was agreed to by 48.31% of the respondents, 61.30% of who were the respondents who administer dietary supplements to children.

Statistical analysis carried out using Spearman’s rank correlation coefficient indicated that the participants of the research had the tendency to perceive DSs as medicinal products and it applied to both people who administer and do not administer DSs to children, as well as to people who consider DSs to be food (Tables S1 and S2, Supplementary Material). This means that the respondents who perceive supplements as drugs, as well as food, tend to see them as having medicinal properties. Respondents who perceive DSs as having medicinal properties are especially likely to perceive supplements as preparations preventing diseases and shortening their duration (ρ-Spearman = 0.49 and 0.48, respectively). It is worth mentioning that the respondents for whom DSs are equivalents of medicines tend not to see that dietary supplements may cause side-effects and interact (ρ-Spearman = 0.14 and 0.16 respectively). An analogical relationship occurs among people who consider administering DSs to be safe for their children’s health. These relationships are strong among both caregivers who administer and those who do not administer dietary supplements to children.

**Figure 3:**
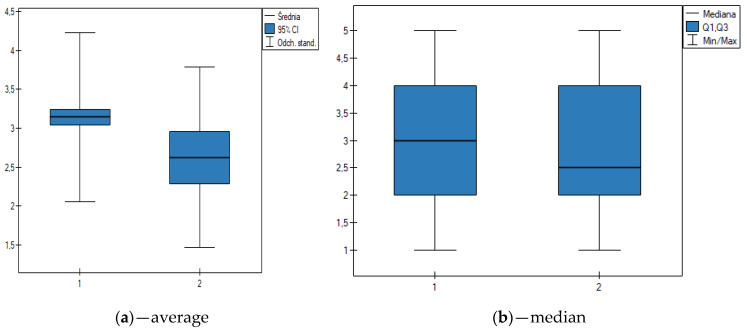
The Mann–Whitney U test for the dependence between gender and the belief that it is safe for children to take dietary supplements if they are administer according to the recommendations (1—women, 2—men, *y*-axis is the 5-point scale where 5 means ‘I strongly agree’ and 1 means ‘I strongly disagree’. Statistically significant differences at *p* ≤ 0.05.

Taking into consideration gender as a grouping factor, on the basis of the Mann–Whitney U test *p* ≤ 0,05, *N* = 532), it can be assumed that there are statistically significant differences between gender and belief that taking DS according to the recommendations is safe. Even though median = 3 allows us to conclude that a significant number of the respondents do not have an opinion on that subject, it is the women who show a higher degree of trust in the safety of taking DS (Figure 3).

**Figure 4:**
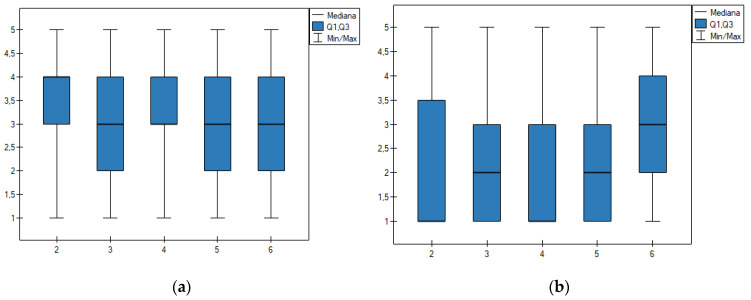
**2**—25–30 y, **3**—20–35 y, **4**—36–40 y, **5**—41–45 y, **6**—46 y and more. Analysis of the age of the respondents and the opinion on the safety of administering DS (**a**) as well as knowledge on the subject of DS falling into the category of food (**b**). The 20–25 age group was not taken into account due to a small sample size.

The knowledge and attitudes of the respondents in regards to administering dietary supplements were also assessed based on the age of the respondents (Figure 4). The results showed that there are statistically significant differences in perceiving DS as preparations, which are safe for health (Figure 4a): respondents aged 26–30 have a higher trust in the safety of DS, median = 4 Kruskal–Wallis test, *p* ≤ 0.05, *N* = 528). The analysis of the understanding of the term dietary supplement allowed to conclude that the respondents aged 26–30, and 36–40 strongly disagree with the statement that dietary supplements are a type of food (median = 1). However, within the other age groups, the median was higher (Figure 4). It can be concluded that the older respondents present a slightly higher general knowledge and awareness on the subject of dietary supplements.

**Figure 5:**
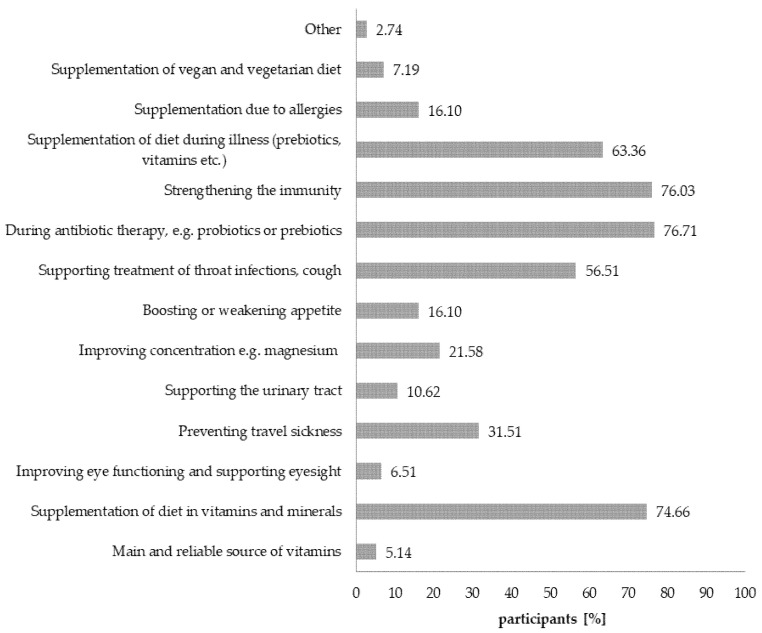
Reasons for administering dietary supplements to children. Values do not add up to 100%, due to the fact that more than one answer was allowed.

Respondents who administer dietary supplements to children (*n* = 292) name the following as the main reasons for doing so: wanting to strengthen a child’s immune system, as support during antibiotic therapy, e.g., probiotics and prebiotics, and to supplement the diet with vitamins and minerals (Figure 5). Other factors mentioned by a part of the population were: supporting the treatment of infections such as flu and cold, and the supplementation of diet during illness (prebiotics, vitamins, etc.) (Figure 5).

**Figure 6:**
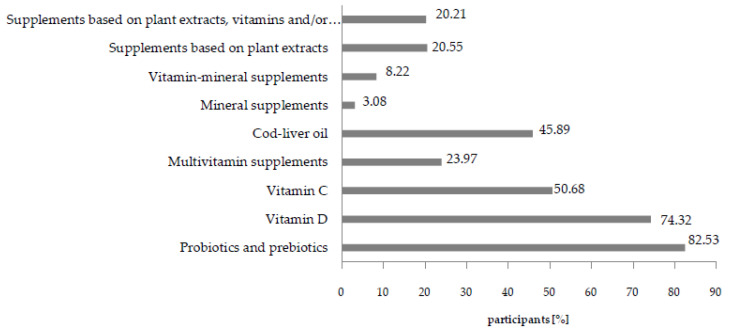
Types of dietary supplements administered to children by caregivers. Percentages add to >100 as participants were able to select >1 reason for us.

The most significant part of the studied population administered probiotics and prebiotics to children (Figure 6). Equally high numbers of caregivers administer vitamin D and C preparations, as well as cod-liver oil.

Concerning administering DSs, caregivers mainly expected that specific ailments their children were suffering from are going to be minimalized (68.84%), and that diet will be supplemented (59.59%). Meanwhile, 26.37% of respondents expected administered supplements to prevent illnesses in children. The remaining answers (other 1.03%) were related to boosting immunity and reducing the number of illnesses. That also suggests that consumers perceive DSs as medicinal products.

As many as 86.64% of respondents (*n* = 292) named pharmacies as the place where they purchase DSs (while 50.68% named them as the only place where they purchase them). Online pharmacies were of less importance and only 11.64% of the respondents named them as the only place where DSs were purchased. Over 33.56% of respondents purchased supplements in stationary and online pharmacies. Remaining sales channels—such as direct sales, large-format stores, and herbal stores—were chosen by 5.12% of the respondents.

**Figure 7:**
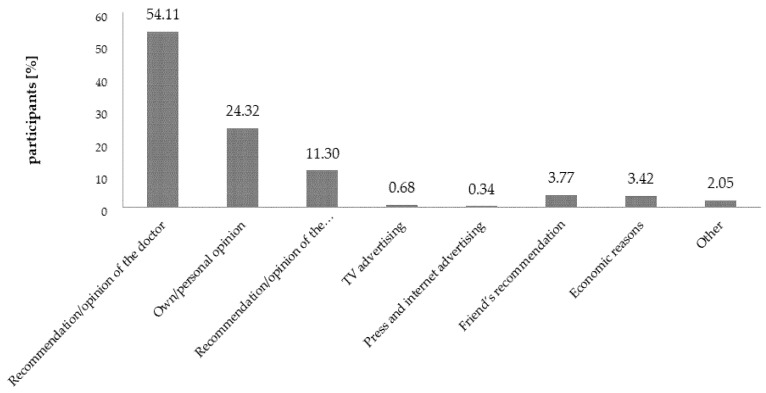
Factors influencing the purchasing decisions made when choosing supplements for children.

Recommendations and opinions of the doctors were a deciding factor influencing the process of selecting supplements, which was confirmed by 54.11% of the respondents (*n* = 292). For 24.32% of them, their own opinion was the deciding factor, and for 11.30%, it was the opinion of the pharmacist (Figure 7). Among the people who declared that they decided based on their own opinions, the overwhelming number (90.14%) of the respondents in that group named Internet materials: articles, blogs, and forums as the source of knowledge in regards to preparations for children. It suggests that to a great extent, it is the media who are shaping the opinions of the consumers.

**Figure 8:**
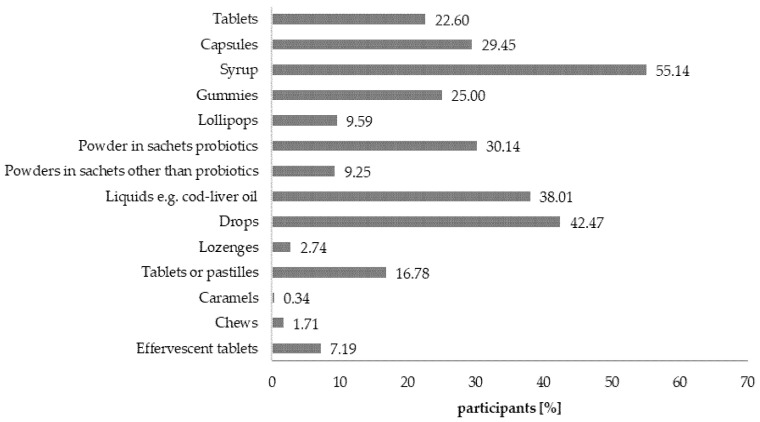
Formulations of dietary supplements intended for children favored by the consumers. Values do not add up to 100%, due to the fact that more than one answer was allowed.

Preferences of respondents in regards to the formulation and composition of the dietary supplements intended for children were studied because the market is filled with supplements in a variety of formulations (Figure 8).

The research determined whether the respondents pay attention to the information included on the labels. It turned out that almost an entire population of people administering dietary supplements to children (*n* = 292), that is 98.97%, read the information prepared by the producers of dietary supplements for children and displayed on the packaging of the products. However, 41.78% of them claimed that this information is not sufficient, and 25.34% did not know if it was sufficient, while 31.85% were satisfied with the amount and the quality of the provided information. Among the respondents, 89.73% read the ingredient lists of preparations intended for children, while 3.77% did not read it if the preparation was recommended by the doctor. Almost all respondents (94.86%, *n* = 292) followed the manufacturer’s instructions regarding the correct dosage of the supplements for children. Nevertheless, 2.05% administered higher doses and 2.40% administered lower doses than recommended. Only two respondents indicated that they do not check such information.

**Figure 9:**
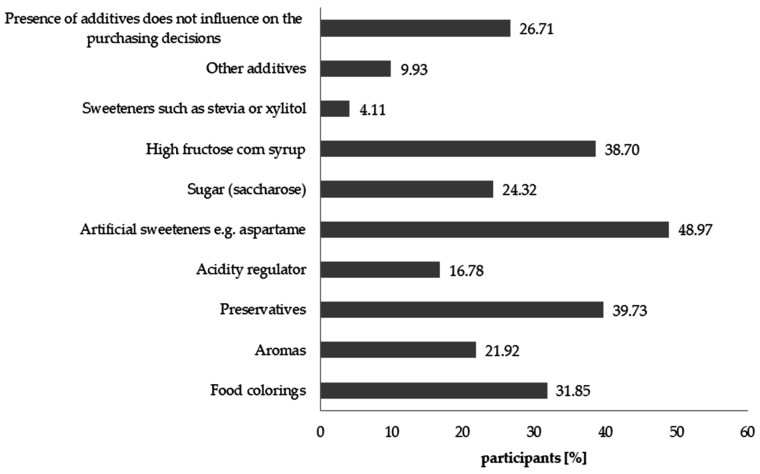
Ingredients of DSs which, when found in the composition of the product for children, may result in respondents deciding not to purchase such product. Values do not add up to 100%, due to the fact that more than one answer was allowed.

The presence of particular raw materials and additives in the composition of the supplement can influence the purchasing decisions of the consumers. Respondents indicated that ingredients such as sweetening agents, preservatives, and high fructose corn syrup could result in them not purchasing the product (Figure 9).

**Figure 10:**
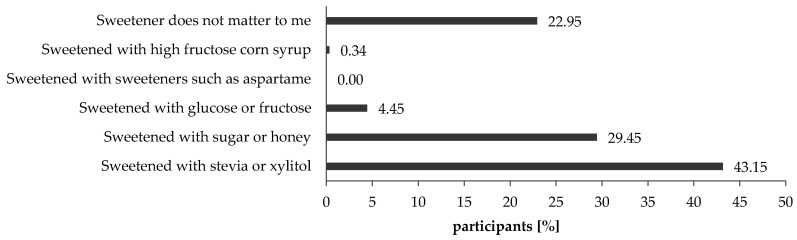
Parent’s preferences regarding the choice of supplements due to the content of sweeteners. Values do not add up to 100%, due to the fact that more than one answer was allowed.

As many as 43.15% of the respondents preferred products sweetened with stevia or xylitol and 29.45% with sugar or honey (Figure 10). Only a small fraction of the respondents indicated that they choose products with glucose, fructose, or high glucose corn syrup. Sweeteners such as aspartame not only did not encourage buying the DS, but their presence in the composition discouraged the consumers from purchasing such products (Figure 9). It is, however, worth mentioning that 26.71% of people claimed that sweetener did not matter to them.

Considering the matter of introducing an obligation to provide a calorific value of the dietary supplements, the opinions of the respondents were divided. The percentage of the respondents who would like for this obligation to be imposed amounted to 45.55%. As many as 43.49% did not have an opinion. As many as 82.88% of the respondents would like to impose on the producers the obligation, to include information on what are the contents of sugars in the product (1.37% wouldn’t like for such obligation to be imposed, 15.75% have no opinion).

It turned out that 20.55% of respondents monitored the amounts of ingested micro- and macro-elements as part of the daily diet and supplementation. However, the remaining 79.45% of the respondents did not analyze the menu of their children to that extent. Among the 32.88% of the respondents who did not control the supply of vitamins and minerals, only 19.79% did not give enriched products to their children.

## 4. Discussion

The last several years have seen an increase in the degree of men’s involvement in raising children. However, women dominate in the aspects associated with care [36,37,38]. A similar relationship has been observed in regards to taking care of children’s health [39]. The distribution of women and men (Table 1) who took part in the research seems to confirm these dependencies.

The percentage of children aged 3 to 12 who take dietary supplements (almost 55%) determined in the course of the research was higher than the percentage that was reported in previous research conducted in Poland. According to this research, it amounted to 40% among children aged 6–12 [40] and 52.3% among children aged 7–12 (*N* = 128) [41]. However, when comparing the selected age ranges (Figure 1), it can be noted that the results are very similar. The percentage of children aged 3–4 taking DSs (65.31%) was higher compared to literature reports (from 30% to 52.2%) [12,14,15]. It was also confirmed that the people who take dietary supplements are transferring their behavioral patterns by also administering them to their children. It was concluded that the caregivers, who administer dietary supplements to their children, show interest in their diet. However, it would be worth considering whether such an analysis of the meal plans is sufficient. Irregularities in the way children are being fed in kindergartens have been proven many times over, e.g., too high consumption (in comparison to recommended amounts) of energy, fats, and carbohydrates [42,43,44]. Similar results were observed in selected schools where the content of carbohydrates in meals ranged from 136% to 300% of the norm for the particular group of students for which the meals were intended. It is well known that BMI is affected by a poorly balanced diet; however, 87% of respondents declared that their children have normal BMI.

Based on the results, it can be concluded that the respondents trust doctors and pharmacists and consider them to be authorities on dietary supplements (Figure 2). The respondents pointed out the importance of online resources. Similar observations concerning the role of the Internet as the source of information were noted [45,46]. The consumers, who base their knowledge of dietary supplements on online resources, are exposed to unfair practices of the dietary supplements’ producers, e.g., using Internet users (employees of the pharmaceutical marketing agencies who create fake social media accounts) who praise the effects of particular products (including their medicinal qualities) [47]. It was reported that online and radio advertisements do not comply with existing legislation [48,49]. Another form of such unfair practices is sponsored ads, that can be found on parenting blogs and websites and the involvement of celebrities in DS advertising which may influence purchasing decisions [49,50]. Moreover, on the online pharmacies’ websites, one can find opinions of caregivers regarding dietary supplements administered to children. However, consumers cannot verify the reliability of such information. This is important because over one-third of the respondents used social media and blogs to search for information about supplements. The analysis of the respondents’ answers allowed us to conclude that the opinion of the doctor and pharmacist is crucial for the caregivers who are choosing the dietary supplements for children aged 3 to 12. However, the participants of the study indicated that online resources are also very significant.

Respondents specified where they usually purchase dietary supplements. Based on their answers, it can be concluded that traditional pharmacies (available to everyone) are the primary sales channel for this type of product. Recently, due to the COVID-19 pandemic resulting in a change of everyday habits related to social interactions, sport, and probably diet, the growing popularity of online sales can be predicted [51].

In the literature, one can find few references related to the possibility of perceiving dietary supplements as medication. Even though the percentage of such answers was not high (6%) (Table 2), it is necessary to consider whether such an image of supplements was suggested to the consumers by the media (TV commercials, internet advertisements, press releases, etc.) and whether pharmacies, which are the primary source of DSs, are not responsible for such perception of these products. In the research conducted by Dickinson et al. [52], 6% of the study group perceived multivitamin supplements as preparations having medicinal properties. In this research, over half of the respondents declared that they have general knowledge about dietary supplements (Table S1, Table 3), while at the same time, less than 25% of the respondents knew that dietary supplements are food products. Even though a small fraction of the study population considered supplements to be drugs (circa 6.5%), over 35% claimed that they can exhibit medicinal properties. Even more people saw their potential to prevent colds and flu. A higher percentage of people administering dietary supplements to children saw their medicinal potential as well as the potential to prevent illness and shorten its duration (Table 3), which is also observed among various consumer groups [17,53]. The answers of the respondents who were allowed to define the term ‘dietary supplement’ allows us to conclude that they perceive supplements as drugs (Table 2). Even though the percentage of such answers was not high, it would be advisable to analyze whether media (TV and internet advertisements, press materials, etc.) are responsible for suggesting such an image of supplements to the consumers and whether pharmacies, which are the primary source of supplements, are the main reason for such perceptions of these products.

Respondents’ views on the subject of the effectiveness and safety of DSs are divided. A higher degree of trust was displayed by a group of people who administer dietary supplements to children (Table 3). It was also observed that women and younger people (Figure 3 and Figure 4) have a tendency to view supplements as safe products as opposed to men. It is important to note that this is not the first time this observation was made [11,17]. Overall, it can be concluded that the results of this research for the group of respondents who administer DSs to children are, to a certain extent, in agreement with the general belief that dietary supplements are safe [54], useful [52], and effective products [55]. The research proved that parents who administer dietary supplements to children show a tendency to have more trust in this type of product than the people who do not do so.

Vitamin–mineral preparations are the most popular type of dietary supplements on the market [56,57]. However, according to the results of the study, the dominant type of products administered to children aged 3–12 are prebiotics and probiotics, which is undoubtedly related to doctors’ recommendations in regards to the protective measures of such products during antibiotic therapy [58]. This was reflected by the answers of over 82% of respondents who declared to have such an attitude (Figure 6).

Consumers’ preferences are partly a reflection of which formulations of DSs are available on the market [59]. Over half of respondents preferred to administer syrups to their children, followed by drops, liquids, and capsules. This is also a result of children’s ability (over 6 years of age) to take/swallow particular products such as tablets or capsules [60]. For that reason, the main formulations of DSs taken by adults are tablets, lozenges, and capsules [61,62], while the main formulations taken by teenagers are inter alia tablets, effervescent powders, and gummies [63]. Based on the research, one can conclude that formulations of dietary supplements that are mimicking conventional foods, especially gummies, are likely to be chosen for children. There is a real risk of children treating such supplements as sweets due to making associations between formulations of sweets and supplements available on the market. However, because of their calorific value (sugar contents) these products can be considered to be unhealthy and give parents (caregivers) a false impression of supplementing the deficiencies in a child’s diet [64]. In reality, it can potentially lead to deterioration of dental health and excessive supply of carbohydrates.

Consumers declared that they adhere to the recommendations concerning the dosage of supplements in the form of conventional foods. It is important in the context of adjusting the supply of macro- and micro-elements to children’s demand for them. Respondents were asked whether they treat DSs as an alternative to sweets in the diet of their children. The results show that 18.84% of the respondents shared this attitude while 81.16% of the remaining respondents did not agree with this statement. The formulation of the supplement is closely related to the presence of food additives, the type and quantity of the sweetener, and therefore with the calorific value of the product [64]. The respondents pointed to additives, which they prefer to avoid in DS intended for children, and the results correlate with those observed previously in Japan [6]. Research on the acceptance of selected additives was already conducted in the 90s [65]. The newest results show that consumers display more trust towards sweeteners than food dyes and that the degree of acceptance is based on the knowledge of legal regulations, preference for natural products, perception of risks and potential benefits [66]. In this research, the opposite was observed. The following factors can be blamed for such results: the target group, for which such products are intended, that is children, and the growing awareness of the respondents in regards to the possible negative impact that such synthetic sweeteners may have on health, moreover, concerns of the respondents in regards to administering to children supplements that contain preservatives and aromas can be linked to the contemporary trend of respondents demanding “clean-label products”, which are products with no artificial additives [67,68]. Nearly 75% of the respondents can identify the sweeteners which they expect to see in dietary supplements for children. Based on that, it can be confirmed that the composition of the supplement, and especially the type of used sweetener and food additives, have an influence on the purchasing decisions of the consumers.

According to the existing EU regulations concerning labeling, consumers can only determine the contents of added sugars based on the ingredient list [69]. This turns out to be a challenge for many consumers because the producers “hide” sweeteners under unfamiliar names [70]. Currently, even well-educated consumers have trouble determining the actual content of sugar added to food [71]. Caregivers declared a need of a better labeling with more precise nutritional information. The goal of introducing additional labeling (that would be devoted to calorific value and the amount of added sugars) would be to improve the quality and the quantity of information intended for the consumers and subsequently simplify the process of choosing healthy products, including dietary supplements for children. Going only by the information on the calorific value without checking the product’s ingredients could potentially lead to the increase in consumption of artificial intense sweeteners products among children.

There is a real possibility of too high supply of vitamins and minerals due to administering a combination of dietary supplements and fortified food to children. This was confirmed by the results of this research. When children are concerned, taking dietary supplements along with enriched foods may lead to exceeding tolerable upper intake level (UL). It was proven that the excessive supply of—e.g., vitamin A, vitamin D, iron or zinc [26,72,73,74,75]—nutrients, which play a crucial role in the functioning of the immune system, may have a synergistic or antagonistic influence on other ingredients found in food. For example, an antagonistic effect was observed between vitamin E and vitamin A [76] as well as vitamin C in combination with more complex multi-vitamin supplements [77].

### Limitations

The subjects of this study did not represent the population of the entire country. All of the data were self-reported, leading to the possibility that the respondents may have misreported some data. The studied group was predominantly female; because of that, an analysis of the influence of gender on particular parameters has not been carried out, as well as the study of the relationship between gender and specific attitudes or behaviors. The same rule was kept in case of a lack of analysis of education (85.55% higher) and place of residence (city 82.89%).

## 5. Conclusions

It was concluded that the caregivers misunderstood the term “dietary supplement”. Despite declaring that they do have general knowledge on the subject, less than 25% of the respondents knew that it is a type of food product. Almost 37% claimed that dietary supplements may have medicinal properties. The results showed that the caregivers knew or partly knew the recommendations of the National Food and Nutrition Institute. However, they did not adhere to them fully. The choice of DS was based on the recommendations of pharmacists and doctors, but they also looked for information online. It was concluded that the caregivers paid attention to the type of sweetening agent used in DS and that it was an important factor influencing their purchasing decisions. It was confirmed that parent’s age and gender influence their opinion (perception of SD) about dietary supplements, and parents who administer dietary supplements to children show a tendency to have more trust in this type of product than the people who do not do so. Individuals were not aware of the risks associated with dietary supplementation. There is also a real risk of children treating dietary supplements as sweets due to associating formulations of sweets with specific formulations of the dietary supplements currently available on the market. Considering the calorific value of such products (the content of sugars) they can be deemed unhealthy. They may also cause the caregivers to have a false impression that DSs take care of supplementing the deficiencies in the diet of a child. In reality, they may cause a deterioration of dental health and too high supply of carbohydrates. To conclude, results show that there is a need to improve parents’ knowledge on the subject of dietary supplements and the conditions of their use.

## Figures and Tables

**Table 1:** Demographic characteristics of the study participants group.

Parameter	Entire Group *N* (%)	Individuals Who Intake DS *n* (%)
Gender:		
Female male	484 (90.98) 48 (9.02)	270 (92.47) 22 (7.53)
Place of residence:		
big city ^1^ town ^2^ village	441 (82.89) 23 (4.32) 68 (12.78)	241 (82.53) 10 (3.42) 41 (14.04)
Education:		
Elementary Vocational Secondary higher	3 (0.56) 11 (2.07) 63 (11.84) 455 (85.53)	1 (0.34) 4 (1.37) 28 (9.59) 259 (88.70)
Age:		
under 20 y/o 20–25 y/o 26–30 y/o 31–35 y/o 36–40 y/o 41–45 y/o 46 and more	0 (0.00) 4 (0.75) 40 (7.52) 182 (34.21) 181 (34.02) 105 (19.74) 20 (3.76)	0 (0.00) 4 (1.37) 21 (7.19) 109 (37.33) 102 (34.93) 49 (16.78) 7 (2.40)
Material situation:		
very good good sufficient bad	111 (20.86) 362 (68.05) 57 (10.71) 2 (0.38)	63 (21.58) 194 (66.44) 34 (11.64) 1 (0.34)
Number of children:		
one two three four five	165 (31.02) 294 (55.26) 63 (11.84) 9 (1.69) 1 (0.19)	89 (30.48) 171 (58.56) 27 (9.25) 4 (1.37) 1 (0.34)

^1^ >20,000 habitants, ^2^ ≤20,000 habitants.

**Table 2:** Selected definitions of dietary supplements given by the respondents *.

Pharmaceuticals	Neutral	Food
Pharmaceuticals containing macro- and micro-elements or vitamins which are taken with the intention of supplementing the diet deficient in such ingredients. It’s a medical product that is not a medicine. Medications aimed at supplementing the diet. Medical products supplementing the everyday diet with vitamins and microelements used in order to supplement or prevent from deficiencies. Medication containing minerals and vitamins. Over the counter medication that has not been tested. ‘Medications’ which list as their ingredients vitamins and minerals essential for maintaining good health and which are supplementing the diet Over the counter medications. Ersatz medications that do not require carrying out tests before they are introduced into the market Medical product the effects of which are not scientifically proven	Tablets Artificial additives Synthetic vitamin equivalent Body strengthener Supplementation Supporter Additive Taking vitamins in the form of tablets Pharmacy preparation described as dietary supplement Preparations that are an additional support for our body Preparations that do not have the status of medication, are not medical products Measures used to support normal growth and development	Supplementation of diet Nutrient that supplements the diet which can be deficient in it Foodstuff which supplements deficiencies Something that supports nutrition Something that supplements diet Something that can replace healthy food Everyday nutrition additive Low-calorie product which replaces food Foodstuffs which contains vitamins, minerals, Omega-3 Fatty Acids etc. Supplementation of daily diet of a child Vitamins and minerals that can serve as a diet replacement Low-calorie product which can replace food Healthy food replacement

* Selected data quoted from the questionnaire filled in by the respondents.

**Table 3:** Evaluation of the knowledge and attitudes of the respondents towards administering dietary supplements.

Nb.	Statement ^1^ (S)	Overall *N* = 532			Administering DSs to Children					
					Yes *n* = 292			No *n* = 240		
		Mean	Median	4 and 5 [%] ^2^	Mean	Median	4 and 5 [%] ^2^	Mean	Median	4 and 5 [%] ^2^
1.	I have general knowledge on the subject of dietary supplements intended for children *	3.48	4	50.75	3.62	4	58.90	3.31	3	40.83
2.	In my opinion dietary supplement is an equivalent of medication	1.56	1	6.39	1.60	1	6.51	1.53	1	6.25
3.	Dietary supplements are foodstuff (type of food)	2.29	2	23.68	2.26	2	21.92	2.29	2	25.83
4.	Dietary supplements (ingredients of dietary supplements) can interact with medication and food consumed by the child	4.20	5	76.50	4.12	4	75.34	4.29	5	77.92
5.	Dietary supplements can cause side effects in children *	4.03	4	71.24	3.86	4	66.44	4.23	5	77.08
6.	Taking dietary supplements in recommended doses is safe for the health of children *	3.10	3	36.84	3.54	4	55.14	2.54	3	14.58
7.	Administering dietary supplements to children can be one way of supplementing their diet *	2.84	3	36.65	3.25	4	50.68	2.36	2	19.58
8.	Taking dietary supplements in an uncontrollable manner (by children) can lead to an overdose of vitamins and minerals or other substances	4.56	5	90.23	4.56	5	90.75	4.56	5	89.58
9.	Dietary supplements intended for children can have medicinal properties *	3.05	3	36.84	3.34	3	47.60	2.73	3	23.75
10.	Selected dietary supplements intended for children are effective in flu and cold prevention *	3.32	3	46.24	3.69	4	62.33	2.90	3	26.67
11.	Taking dietary supplements (by children) during illness can shorten its duration.	3.36	3	48.31	3.67	4	61.30	3.06	3	32.50

^1^ Multiple-choice statements using a five-point scale, where 1 meant ‘strongly disagree’, 2—‘I rather disagree’, 3—‘I have no opinion’, 4—‘I rather agree’, 5—‘I definitely agree’. ^2^ Percentage of responses 4 and 5, DSs dietary supplements, * Statistically significant difference at *p* ≤ 0.05 (The Mann–Whitney U test).

## Supplementary Materials

The following are available online at https://www.mdpi.com/2072-6643/12/10/3076/s1, Table S1: The value of the ρ-Spearman coefficient for the relationship between individual attitudes of caretakers (*N* = 532), Table S2: The value of the ρ-Spearman coefficient for the relationship between individual attitudes of caretakers who administer dietary supplements to children (*N* = 292).
