# Supplementary material for: What Do Polish Parents and Caregivers Think of Dietary Supplements for Children Aged 3–12?
Source: Nutrients. 2020 Oct 9;12(10):3076. doi: 10.3390/nu12103076 (PMC7600402; doi:10.3390/nu12103076)
Supplement: Supplementary file 1 [file nutrients-12-03076-s001.pdf]

Table S 1. The value of the ϱ-Spearman coefficient for the relationship between individual attitudes of caretakers (N=532)

Statement (S)		1	2	3	4	5	6	7	8	9	10	11
		I have general knowledge on the subject of DS intended for children	In my opinion dietary supplement is an equivalent of medication	DS are foodstuff (type of food)	DS (ingredients of DS) can interact with medication and food consumed by the child	Dietary supplements can cause side effects in children	Taking DS in recommended doses is safe for the health of children	Administering DS to children can be one way of supplementing their diet	Taking DS in an uncontrollable manner (by children) can lead to an overdose of vitamins and minerals or other substances	Dietary supplements intended for children can have medicinal properties	Selected dietary supplements intended for children are effective in flu and cold prevention	Taking DS (by children) during illness can shorten its duration.
1	I have general knowledge on the subject of DS intended for children	x	-	-	0,10		0,16	0,11	0,11		0,10	-
2	In my opinion dietary supplement is an equivalent of medication	-	x	-	-0,16	-0,14	-	-	-	0,25	0,19	0,15
3	Dietary supplements are foodstuff (type of food)	-	-	x	0,09		-	0,13	-	0,10	-	-
4	DS (ingredients of DS) can interact with medication and food consumed by the child	0,10	-0,16	0,09	x	0,55	-0,13	-	0,40	-	-0,11	-
5	Dietary supplements can cause side effects in children	-	-0,14	-	0,55	x	-0,23	-0,17	0,37	-	-0,14	-0,12
6	Taking dietary supplements in recommended doses is safe for the health of	0,16	-	-	-0,13	-0,23	x	0,53	-	0,33	0,47	0,39

	children											
7	Administering DS to children can be one way of supplementing their diet	0,11	-	0,13	-	-0,17	0,53	x	-	0,34	0,43	0,39
8	Taking DS in an uncontrollable manner (by children) can lead to an overdose of vitamins and minerals or other substances	0,11	-	-	0,39	0,37	-	-	x	-	-	-
9	DS intended for children can have medicinal properties	-	0,25	0,10	-	-	0,33	0,34	-	x	0,49	0,48
10	Selected dietary supplements intended for children are effective in flu and cold prevention	0,10	0,19	-	-0,11	-0,14	0,47	0,43	-	0,49	x	0,69
11	Taking DS (by children) during illness can shorten its duration.	-	0,15	-	-	-0,12	0,39	0,39	-	0,48	0,69	x

„-”no statistically significant relationship, p>0,05

Table S 1. The value of the ϱ-Spearman coefficient for the relationship between individual attitudes of caretakers who administer dietary supplements to children (N=292)

Statement (S)		1	2	3	4	5	6	7	8	9	10	11
		I have general knowledge on the subject of DS intended for children	In my opinion dietary supplement is an equivalent of medication	Dietary supplements are foodstuff (type of food)	DS (ingredients of DS) can interact with medication and food consumed by the child	Dietary supplements can cause side effects in children	Taking dietary supplements in recommended doses is safe for the health of children	Administering dietary supplements to children can be one way of supplementing their diet	Taking DS in an uncontrollable manner (by children) can lead to an overdose of vitamins and minerals or other substances	Dietary supplements intended for children can have medicinal properties	Selected dietary supplements intended for children are effective in flu and cold prevention	Taking dietary supplements (by children) during illness can shorten its duration.
1	I have general knowledge on the subject of DS intended for children	x	-	0,12	-	-	0,24	0,19	-	0,20	0,12	-
2	In my opinion dietary supplement is an equivalent of medication	-	x	-	-0,13	-0,12	-	-	-	0,26	0,12	-
3	Dietary supplements are foodstuff (type of food)	0,12	-	x		-	-	-	-	0,12	-	-
4	DS (ingredients of DS) can interact with medication and food consumed by the child	-	-0,13	-	x	0,54	-0,13	-	0,40	-	-0,16	-
5	Dietary	-	-0,12	-	0,53	x	-0,15	-	0,36	0,14	-0,12	-

	supplements can cause side effects in children*											
6	Taking dietary supplements in recommended doses is safe for the health of children	0,24	-	-	-0,13	-0,15	x	0,48	-	0,22	0,39	0,33
7	Administering DS to children can be one way of supplementing their diet	0,19	-	-	-	-	0,48	x	-	0,26	0,36	0,30
8	Taking DS in an uncontrollable manner (by children) can lead to an overdose of vitamins and minerals or other substances	-	-	-	0,40	0,36	-	-	x	-	-	-
9	DS intended for children can have medicinal properties*	0,20	0,26	-	-	0,14	0,22	0,26	-	x	0,42	0,41
10	Selected DS intended for children are effective in flu and cold prevention	0,12	0,12	-	-0,16	-0,12	0,39	0,36	-	0,42	x	0,59

11	Taking DS (by children) during illness can shorten its duration.	-	-	-	-	-	0,33	0,30	-	0,41	0,59	x

„-"no statistically significant relationship p>0,05
